# IgG1/IgE ratio is relevant for the protective effect of an alum-adjuvated allergen vaccine in a murine model of local allergen challenge

**DOI:** 10.1186/1939-4551-8-S1-A42

**Published:** 2015-04-08

**Authors:** Wendy Ramírez González, Roxana Samalea, Damarys Toralba Averoff, Alexis Labrada, Alain Morejón, Oliver Perez, Juan Francisco Infante

**Affiliations:** 1 National Center of Bioproducts, Cuba; 2 University of Medical Sciences of Havana, Cuba; 3 Finlay Institute, Cuba

## Background

The new anti allergic vaccine is based on purified allergens from *Dermatophagoides siboney* and the combination adjuvant containing a TLR-4 ligand and alum. We propose that IgG1/IgE ratio is effective to assess the clinical efficacy of this experimental vaccine. Aim: To evaluate the immunogenicity of formulation variants of a *D. siboney* adjuvanted vaccine with varying levels of allergen adsorption on to alum.

## Methods

Vaccine formulations with different allergen adsorption levels were obtained by variation of phosphate ions and alum hydroxide content. In a preventative experimental setting, BALB/c mice were treated subcutaneously with 3 doses of each vaccine variant seven days apart. Further, mice were subjected to aerosol allergen challenge. The allergen-specific antibody response was assessed by determining serum levels of IgE, IgG, IgG1 and IgG2a by ELISA, as well the local allergic response by cytokine levels in broncho-alveolar lavage.

## Results

All variants induced allergen-specific IgG1 and IgG2a antibodies after three immunization doses. The low alum variant showed significant reduction of IgE, whereas the variant with the highest adsorption level (lowest phosphate content) showed significant increase of IgG1. In contrast IgG2a antibodies were not affected by allergen adsorption. A significant increase of the IgG1/IgE ratio was observed for all variants in challenged mice as compared to Th2 controls, with preponderance of the variants with reduced alum content and highest adsorption level. Cytokine levels in BAL indicated a mild but significant shift from Th2 to Th1/Th0 pattern. Histological examination of lungs showed a diminished allergic inflammatory response in vaccinated mice. Reduced size of granulomes at injection site was noted in subjects vaccinated with the low alum formulation variant.

## Conclusions

Serum IgG1/IgE ratio can be indicative of a protective immunogenicity in a murine model of respiratory allergy. Higher allergen adsorption on to alum could be related to a more effective presentation pathway, or to the function of IgG1 as blocking antibody.

